# Experimental virus evolution in cancer cell monolayers, spheroids, and tissue explants

**DOI:** 10.1093/ve/veab045

**Published:** 2021-05-06

**Authors:** Ahmed Al-Zaher, Pilar Domingo-Calap, Rafael Sanjuán

**Affiliations:** Institute for Integrative Systems Biology (I2SysBio), Universitat de València-CSIC, C/ Catedrático Agustín Escardino 9, València 46980, Spain

**Keywords:** experimental evolution, oncolytic virus, vesicular stomatitis virus

## Abstract

Viral laboratory evolution has been used for different applications, such as modeling viral emergence, drug-resistance prediction, and therapeutic virus optimization. However, these studies have been mainly performed in cell monolayers, a highly simplified environment, raising concerns about their applicability and relevance. To address this, we compared the evolution of a model virus in monolayers, spheroids, and tissue explants. We performed this analysis in the context of cancer virotherapy by performing serial transfers of an oncolytic vesicular stomatitis virus (VSV-Δ51) in 4T1 mouse mammary tumor cells. We found that VSV-Δ51 gained fitness in each of these three culture systems, and that adaptation to the more complex environments (spheroids or explants) correlated with increased fitness in monolayers. Most evolved lines improved their ability to suppress β-interferon secretion compared to the VSV-Δ51 founder, suggesting that the selective pressure exerted by antiviral innate immunity was important in the three systems. However, system-specific patterns were also found. First, viruses evolved in monolayers remained more oncoselective that those evolved in spheroids, since the latter showed concomitant adaptation to non-tumoral mouse cells. Second, deep sequencing indicated that viral populations evolved in monolayers or explants tended to be more genetically diverse than those evolved in spheroids. Finally, we found highly variable outcomes among independent evolutionary lines propagated in explants. We conclude that experimental evolution in monolayers tends to be more reproducible than in spheroids or explants, and better preserves oncoselectivity. Our results also suggest that monolayers capture at least some relevant selective pressures present in more complex systems.

## 1. Introduction

Experimental virus evolution has been used for investigating basic evolutionary processes under controlled laboratory conditions, as well as for clinical applications. These applications include the production of live attenuated vaccines (Martín and Minor 2002), analysis of vaccine reversion to virulent phenotypes (Stern et al. 2017), modeling viral emergence in the laboratory (Elena, Fraile, and García-Arenal 2014; Morley, Mendiola, and Turner 2015; Pepin et al. 2019), predicting the appearance of drug resistances (Dickinson et al. 2014), and optimization of therapeutic viruses (Sanjuán and Grdzelishvili 2015; Zainutdinov et al. 2019). For experimental virus evolution to provide useful results, though, laboratory conditions should reproduce relevant selective pressures found in nature (Geoghegan and Holmes 2018: 40). In plant and bacterial viruses, such studies can be easily performed *in vivo*, which complex virus–host interactions to be investigated (Bull 2008; Elena et al. 2008; Hajimorad et al. 2011; Elena 2017). However, for both practical and ethical reasons, few studies have undertaken evolutionary experiments with animal viruses *in vivo* (Jerzak et al. 2008; Keleta et al. 2008; Kubinak et al. 2012; Beier, Hermiston, and Mumberg 2013; Dalkara et al. 2013; Hall et al. 2017). Animal viruses are typically evolved in cell line monolayers, which represent a highly simplified system. In many cases, these cultures are highly permissive to viral infection, since they exhibit little or no antiviral responses and lack spatial structure. Such permissivity is an advantage for viral amplification and stock production, but might limit the relevance of experimental evolution studies. Here, we set out to compare the evolutionary trajectories of a model virus in three cell culture systems of increasing complexity: standard cell monolayers, tridimensional cell masses (spheroids), and tissue explants.

We performed these experiments in the context of oncolytic viruses. Genetic engineering has allowed the development of viruses that preferentially target and destroy tumor cells. Over a hundred oncolytic viruses have entered clinical trials, but in most cases their efficacy has been modest, and very few have been approved for clinical use (Lawler et al. 2017; Lemay et al. 2018; Zheng et al. 2019; Mondal et al. 2020). The therapeutic effect of these viruses is not determined solely by the direct killing of tumor cells, but also by immune stimulatory effects associated with viral infection, and by the destruction of tumor-associated structures such as connective tissue and vasculature (Breitbach, Lichty, and Bell 2016; Bai et al. 2019; Lemos de Matos, Franco, and McFadden 2020). This suggests that *in vitro* testing and optimization of oncolytic viruses should work best in the context of complex cellular environments and virus–host interactions. Spheroids have been used as models in anticancer drug screening because they contain cells with varying levels of nutrient supply and allow examining the ability of these drugs to penetrate inside solid tumors (Kiyohara et al. 2016; Hamilton and Rath 2019). Explants from tumors produced in animals also display these features, and include additional elements such as tumor-associated cell types, meaning that they should more closely reproduce the microenvironment and cellular functionality found *in vivo* (Collins et al. 2020; Powley et al. 2020).

Virus–host interactions are complex, sometimes limiting our ability to obtain efficacious oncolytic viruses through rational design. This limitation is further accentuated by the fact that tumor cells are a highly heterogeneous, evolving target (McGranahan and Swanton 2017), complicating the design of therapeutic viruses for each specific cell type. Directed evolution offers an alternative approach, in which selection can be harnessed to adapt viruses to specific target cells even if the underlying mechanisms are not initially understood (Sanjuán and Grdzelishvili 2015; Zainutdinov et al. 2019). Yet, the number of directed evolution studies with oncolytic viruses is relatively small and, surprisingly, most of these studies have been performed with DNA viruses, particularly adenoviruses (Yan et al. 2003; Subramanian, Vijayalingam, and Chinnadurai 2006; Gros et al. 2008; Kuhn et al. 2008; Puig-Saus et al. 2012; Wechman et al. 2016), despite the fact that RNA viruses exhibit higher mutation rates (Sanjuán and Domingo-Calap 2016) and, hence, should lend themselves more easily to evolutionary optimization.

As a model system, we used vesicular stomatitis virus (VSV), a negative-stranded 11.2 kb RNA virus of the family *Rhabdoviridae*. VSV has been used previously for basic experimental evolution studies (Elena and Sanjuán 2007) and as a platform for oncolytic virus design (Hastie and Grdzelishvili 2012; Felt and Grdzelishvili 2017). VSV oncoselectivity resides essentially in the viral matrix protein M, which functions as a suppressor of cellular gene expression by inhibiting mRNA nuclear export. As a result, infected cells fail to mount an innate immune response against the virus (Faul, Lyles, and Schnell 2009; Rieder and Conzelmann 2009). This function of the M protein can be impaired by mutations at specific residues, of which methionine 51 is the best characterized (Kopecky, Willingham, and Lyles 2001). Such mutants show a highly attenuated phenotype in normal cells but not in tumor cells with deficient innate immunity, providing oncoselectivity (Stojdl et al. 2000, 2003). Based on this principle, an oncolytic VSV was obtained by deleting M codon 51 (VSV-Δ51) (Hastie and Grdzelishvili 2012). This mutant infects some cancer cell types efficiently, but remains poorly infectious in other cell types, particularly those with functional innate immunity (Escobar-Zarate et al. 2013; Moerdyk-Schauwecker et al. 2013; Bishnoi et al. 2018).

Previous studies have used VSV for oncolytic virus-directed evolution. In one study, a single-chain antibody against the Her2/neu receptor (ErbB2) was cloned in the viral genome to confer tropism toward tumoral cells, but the engineered VSV showed poor replication in ErbB2-expressing mammary cancer cells, and directed evolution was used to improve this trait (Gao et al. 2006). In another work, wild-type (WT) VSV was evolved in human glioblastoma cells with the aim of promoting selective attachment to these cells and replication (Wollmann, Tattersall, and van den Pol 2005). The resulting virus was later shown to be also effective against other types of tumor cells (Wollmann et al. 2013). VSV was also passaged serially in mouse embryonic fibroblasts (MEFs) deficient for the p53 gene to investigate possible mechanisms of tumor selectivity (Garijo et al. 2014). Recently, directed evolution was employed to improve the ability of a VSV-Δ51 derivative to infect pancreatic ductal adenocarcinoma cells (Seegers et al. 2020).

Here, we have compared the evolution of VSV-Δ51 in cell monolayers, spheroids, and explants of the 4T1 mouse mammary carcinoma, a cancer type against which this virus shows poor efficacy (Garijo et al. 2014). We found that VSV-Δ51 gained fitness in each of the three culture systems. Furthermore, evolution in spheroids or explants tended to concomitantly increase viral fitness in monolayers, revealing common selective pressures such as the need to block interferon (IFN)-mediated innate immunity. Yet, evolutionary outcomes specific to each culture system were also found. Importantly, viruses evolved in spheroids tended to show higher fitness in non-tumoral mouse cells than those evolved in monolayers, suggesting that viral optimization through directed evolution should better preserve oncoselectivity when performed in monolayers.

## 2. Methodology

### 2.1 Viruses and cells

VSV-Δ51 was kindly provided by Valery Grdzelishvili (University of North Carolina). This virus belongs to the Indiana VSV serotype and carries a deletion of methionine 51 in the M protein, as well as a Green fluorescent protein (GFP) reporter inserted between the G and L genes. A WT VSV was used as a control for IFN assays. The 4T1 mammary carcinoma cell line was obtained from the American Type Culture Collection (ATCC). BHK-21 baby hamster kidney fibroblasts (from ATCC) were used for plaque assays. MEFs were obtained from Dr Carmen Rivas (Universidad de Santiago de Compostela). Neuro2a cells were provided by Dr José Manuel García-Verdugo (Universitat de València). Cells tested negative for mycoplasma by PCR and were cultured in Dulbecco’s modified Eagle’s Medium (DMEM) supplemented with 10 per cent fetal bovine serum (FBS) in a humidified incubator at 37°C with 5 per cent CO_2_.

### 2.2 Spheroid production

Approximately 7,000 4T1 cells were seeded in 100 µl of DMEM supplemented with 10 per cent FBS, poured into ultra-low-attachment round-bottom culture wells in 96-well plates, and incubated for 16 h to allow spheroid formation. Each well contained a single spheroid. In order to check the tridimensional nature of the spheroids, cells were washed with PBS buffer three times, fixed with paraformaldehyde 4 per cent, maintained in PB azide, cryoprotected in 30 per cent sucrose solution overnight, embedded in optimal cutting temperature solution, frozen, and used for obtaining 10 µm sections in a cryostat. Samples were placed on slides, stained with DAPI, washed three times with PB, protected with Fluorsave, and imaged under a conventional fluorescence microscope.

### 2.3 Explant production

Tumors were established by implanting of 2 × 10^6^ 4T1 cells into the flanks of 7-week-old BALB/C mice. When tumors reached 1,000 mm^3^, animals were sacrificed and tumors were extracted. To obtain explants, the extracted tumors were washed with PBS, cut manually with a surgical blade, and placed in dishes filled with Hanks balanced salt solution. Explants were distributed in cryotubes containing DMEM with 5 per cent DMSO and 10 per cent FBS, and frozen at −80°C. To analyze their viability, explants were thawed and cultured for 24 h in DMEM containing 10 per cent FBS, stained with cell viability indicator Cytotox red, and imaged.

### 2.4 Titration by the plaque assay

BHK-21 monolayers cultured in six-well plates were inoculated with 100 µl of viral suspensions, incubated at 37°C in a 5 per cent CO_2_ for 45 min, and overlaid with 2 ml of DMEM supplemented with 10 per cent FBS and 0.5 per cent (w/v) agar. After 20 h, monolayers were fixed with 2 ml of 10 per cent formaldehyde for 15 min, stained with 2 per cent crystal violet in 10 per cent formaldehyde, and plaques were counted manually.

### 2.5 Monolayer infection

Cells were seeded in six-well plates at a density of 10^5^ cells/well 24 h prior to inoculation. Then, 100 µl containing 10^4^ plaque-forming units (PFU) were used for inoculating cultures and, after a 45-min incubation, cultures were overlaid with 2 ml of DMEM supplemented with 10 per cent FBS. At 22–24 h post-inoculation (hpi), media were collected, titrated by the plaque assay, and used as inoculum for the following transfer.

### 2.6 Spheroid infection

Spheroids were seeded as indicated and, after 16 h, 100 µl containing 10^4^ PFU were added. Culture media were collected at 23 hpi. Since titers at endpoint were on the order of 10^6^ PFU/ml, the dilution required for the following transfer was on the order of 1/10. As a result, a significant amount of IFN and other cytokines could be carried forward between transfers, inhibiting infection from the outset. To avoid this, the collected medium was spun to remove large debris, resuspended in 150 µl of DMEM supplemented with 10 per cent FBS and then centrifuged at 30,000 *g* for 1 h 30 min to pellet the virus and remove the cytokine-containing supernatant. The viral pellet was resuspended in 10 µl of DMEM, titrated, and used for the following transfer.

### 2.7 Explant infection

Two explants were placed in a well of a twelve-well plate with 1 ml of culture medium and, after 16 h, 100 µl containing 10^4^ PFU were added. At 22 hpi, explants were processed in a Precellys homogenizer and the homogenate was spun at 10,000 *g* for 10 min to remove large debris. To avoid carryover of cytokines between serial transfers, the virus-containing supernatant was centrifuged at 32,000 *g* for 2 h. The virus-containing pellet was resuspended in 10 µl of DMEM, titrated, and used for the following transfer.

### 2.8 Flow cytometry

Explants were disaggregated under agitation with trypsin-EDTA, washed twice with PBS 1X by centrifugation (1,500 rpm, 5 min), resuspended in 1 ml 4 per cent paraformaldehyde for fixation, and incubated overnight at 4°C. The fixator was removed and washed by centrifugation (2,000 rpm, 10 min), cells were resuspended in PBS at a density of ca. 10^6^ cells/ml, and 10^4^ events per sample were analyzed in a Becton Dickinson LSRFortessa flow cytometer equipped with a 561-nm laser for FITC-A (GFP) detection.

### 2.9 Automated real-time fluorescence microscopy

Infections were performed as indicated, plates were placed in an IncuCyte automated microscope located inside an incubator, and images were acquired every 2 h using the phase contrast and green channels with a 4× objective. Images were analyzed with built-in software. For this, masks were defined for each channel using a set of representative images, and images were binarized to determine the area occupied by total cells (phase contrast) and infected cells (GFP positive). Background fluorescence was corrected using the Top-Hat method. Area data were exported and used to fit a logistic growth model of the form $A_{t}=\frac{A_{\max}}{1+e^{c-rt}},\;$where *t* is infection time, *A_t_* is the percent area occupied by GFP-positive cells, *A*_max_ is the maximum *A_t_* value, *r* is the exponential infection spread rate, and *c* sets initial conditions. The model was fit to the data by non-linear least-squares regression. Each experimental replicate was individually used for inferring growth parameters, such that *n* = 3 values were obtained for each parameter. In all cases, the goodness-of-fit of the model was *r*^2^ > 0.95.

### 2.10 IFN quantitation by ELISA

Monolayers of 4T1 cells were inoculated at a multiplicity of 3 PFU/cell and, at 16 hpi, 100 μl supernatant was collected, diluted 1:5, and incubated in a 96-microtiter plate with standards supplied by the manufacturer (Mouse IFN beta SimpleStep ELISA Kit, Abcam). Samples were processed following manufacturer’s instructions and absorbance at 450 nm was quantified in a plate reader.

### 2.11 Sample preparation for Illumina deep sequencing

RNA was extracted from viral supernatants using ZR Viral RNA Kit (Zymo Research). The viral genomic RNA was reverse transcribed in three fragments using the following sequence specific plus-strand primers: 5′-CCATTATTATCATTAAAAGGCTC-3′ (sites 16–38), 5′-GGAAAGCATTGAACAAACG-3′ (sites 3,404–3,422), and 5′-GCTTGCACAGTTCTACTTTC-3′ (sites 8,093–8,112). Reverse transcription was performed from 2 μl purified RNA using SuperScript IV First-strand Synthesis System (ThermoFisher Scientific) following manufacturer’s recommendations. Each of the three cDNA products was subject to 35 cycles of PCR using the plus-strand primers 5′-CCATTATTATCATTAAAAGGCTC-3′ (sites 16–38), 5′-CTACCACAGAAAGGGAACTG-3′ (sites 4,174–4,193), and 5′-CAGATCCCGTAACAGAAAGT-3′ (sites 8,195–8,214), and the minus-strand primers 5′-AGCTAAGATGAAGATCGGAG-3′ (sites 4,323–4,304), 5′-GTCTTTAACAAGTTCGCTGG-3′ (sites 8,393–8,374), 5′-ACGAAGACCACAAAACCAG-3′ (sites 11,922–11,904). PCR was done with Phusion High-Fidelity DNA Polymerase (ThermoFisher Scientific) following manufacturer’s instructions. PCR products were verified by agarose gel electrophoresis, purified with the DNA Clean and Concentrator kit (Zymo Research), and quantified by spectrometry. The PCR products of each sample were mixed equimolarly for Illumina sequencing in an MiSeq machine with paired-end libraries.

### 2.12 Deep-sequencing analysis

Evaluation of NGS sequencing FastQ files quality was done with FastQC 0.11.7 (http://www.bioinformatics.babraham.ac.uk/projects/fastqc/). Removal of sequence adapters was done by cutting the first ten nucleotides and the last two nucleotides of each read with Cutadapt (https://cutadapt.readthedocs.io). Then, reads were trimmed with FASTQ Quality Filter (http://hannonlab.cshl.edu/fastx_toolkit/) and Prinseq-lite 0.20.4 by quality (Q30), length (200 nucleotides), and sequencing artifacts (duplications, Ns). The genome of the founder was used for mapping and SNP calling with ViVan 0.43 (Isakov et al. 2015).

## 3. Results

### 3.1 VSV experimental evolution in different culture systems

**Figure 1.**
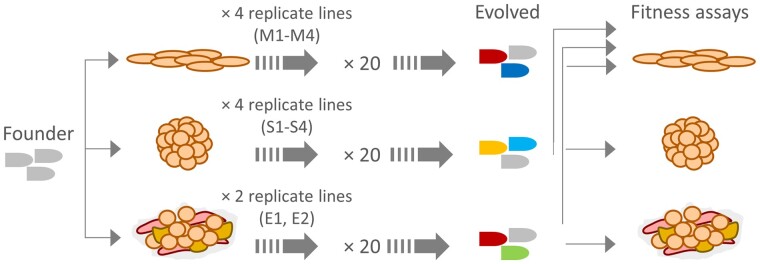
Schematic view of the experimental design. The founder virus (VSV-Δ51-GFP) was transferred twenty times in 4T1 monolayers (four replicate lines), spheroids (four replicate lines), or explants (two replicate lines). Then, the founder and evolved lines were assayed for fitness in their respective evolution environments, as well as in 4T1 monolayers. In addition, all lines were assayed for fitness in MEF monolayers to assess oncoselectivity, sequenced to investigate the genetic basis of adaptation, and tested for their ability to suppress β-IFN secretion.

We performed twenty serial transfers of a VSV-Δ51 encoding the GFP reporter in 4T1 monolayers, spheroids, or explants (Fig. 1). We carried out four independent experimental evolution replicates in monolayers and spheroids, and two replicates in explants. In each passage, cultures were inoculated with approximately 10^4^ PFU, such that there were enough PFU to promote the emergence of high-fitness variants but the multiplicity of infection was kept below 1.0 PFU/cell to prevent the accumulation of deleterious mutations (Sanjuán and Grdzelishvili 2015). In preliminary assays, we found that VSV-Δ51-GFP produced (2.9 ± 0.5) × 10^9^, (9.4 ± 2.8) × 10^5^, and (9.0 ± 1.7) × 10^5^ PFU in monolayers, spheroids, and explants after 22 h of incubation, respectively. Longer incubation times did not yield higher titers in any of the three systems. Hence, as expected, cell monolayers offered a much more permissive environment for viral infection than spheroids or explants. In these initial assays, we verified that spheroids were constituted by a solid tridimensional mass of cells, of which VSV-Δ51-GFP only succeeded to infect the outermost layers (Supplementary Fig. S1). Limited viral spread was also found in explants, despite the fact that most cells appeared to be viable (Supplementary Fig. S2).

### 3.2 Analysis of environment-specific viral adaptation

**Figure 2.**
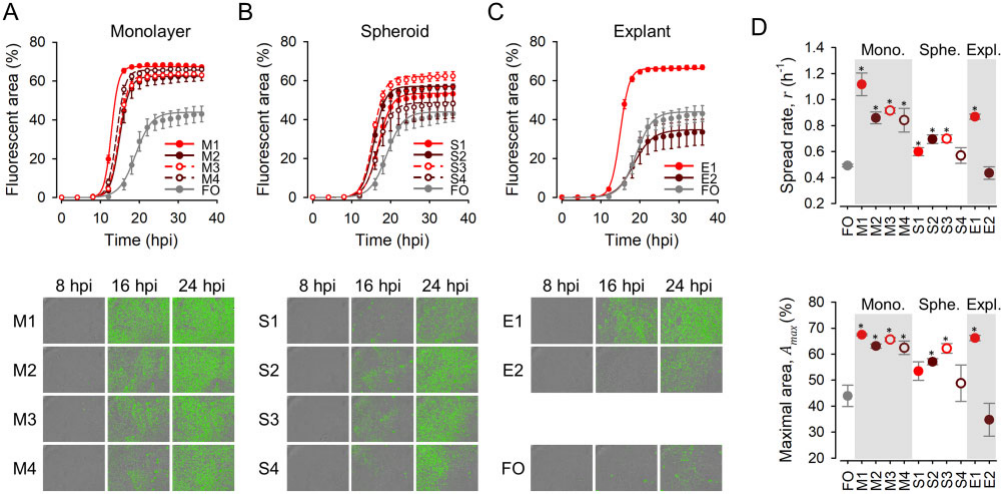
Viral fitness assays in 4T1 monolayers. (A–C) Top: Growth curves obtained by automated real-time fluorescence microscopy for M1–M4 monolayer-evolved viruses, S1–S4 spheroid-evolved viruses, E1–E2 explant-evolved viruses, and the founder virus (FO), respectively. Cells were seeded in six-well plates at a density of 10^5^ per well and, after 24 h, cells were inoculated with approximately 10^4^ PFU. Lines indicate the predicted values obtained from a logistic growth model. Error bars correspond to the SEM (*n* = 3 replicates). Bottom: representative images obtained at different time points. (D) Spread rate (*r*) and maximal infected area (*A*_max_) obtained from the logistic growth model. Asterisks indicate values significantly different from that of the founder virus (*t*-test: *P *<* *0.05).

We then set out to test whether the evolved lines showed higher fitness that the founder virus in their respective environments. Fitness was defined as the number of infected cells. For this, we obtained growth curves in which the area of GFP-positive cells was quantified at regular intervals using real-time automated fluorescence microscopy. The four lines evolved in 4T1 monolayers (M1–M4) showed clearly higher GFP signal than the founder virus when tested in monolayers (Fig. 2A). To analyze these data quantitatively, we used a logistic growth model, $A_{t}=\frac{A_{\max}}{1+e^{c-rt}}$, where *A*_max_ is the maximal GFP-positive area and *r* the viral spread rate. This showed that M1–M4 evolved significantly higher *A*_max_ and *r* values than the founder (*n* = 3 replicates; *t*-tests: *P* < 0.020; Fig. 2D).

**Figure 3.**
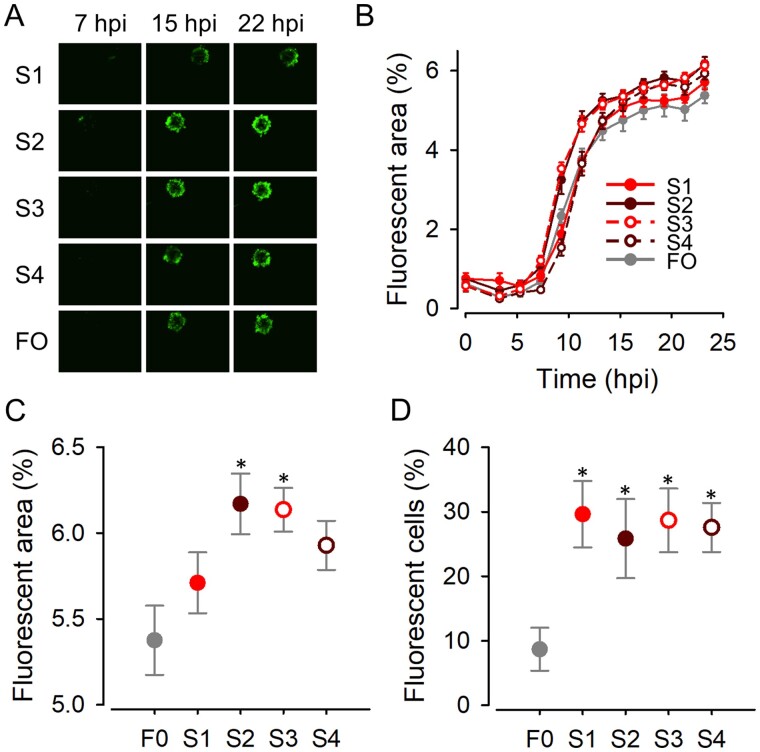
Viral fitness assays in 4T1 spheroids. (A) Representative images of spheroids infected with each virus. (B) Growth curves obtained by automated real-time fluorescence microscopy for S1–S4 and founder viruses. Lines are depicted to connect different time points only (no model fit). Error bars correspond to the SEM (*n* = 6 replicates). (C) Percent area occupied by GFP-positive cells at 22 hpi. (D) Percent fluorescent cells obtained after disaggregating spheroids. All spheroids were inoculated with 10^4^ PFU. Error bars indicate the SEM (*n* = 5 replicates). Asterisks show values significantly different from that of the founder virus (*t*-test: *P *<* *0.05).

We also used real-time automated fluorescence microscopy to test the four lines evolved in spheroids (S1–S4) and the founder virus. When analyzed directly by fluorescence microscopy, the growth curves obtained in spheroids were similar for all five viruses (Fig. 3A and B), although two of the spheroid-evolved lines (S2, S3) appeared to show slightly greater GFP-positive areas in late time points. Direct comparison of the apparent fluorescent areas at 22 hpi confirmed this (*n* = 6; *t*-tests: *P* < 0.015; Fig. 3C). Since imaging of 3D structures is problematic, though, we disaggregated the spheroids at endpoint and counted the percentage of GFP-positive cells. This revealed that the evolved viruses S1–S4 infected approximately three times more cells (ranging from 25.5 ± 6.1 per cent to 29.6 ± 5.1 per cent) than the founder at 22 hpi (8.7 ± 3.3 per cent; *n* = 5; *t*-tests: *P* < 0.048; Fig. 3D). Therefore, serial transfers in spheroids improved viral fitness, although this effect was not obvious by directed imaging of spheroids.

**Figure 4.**
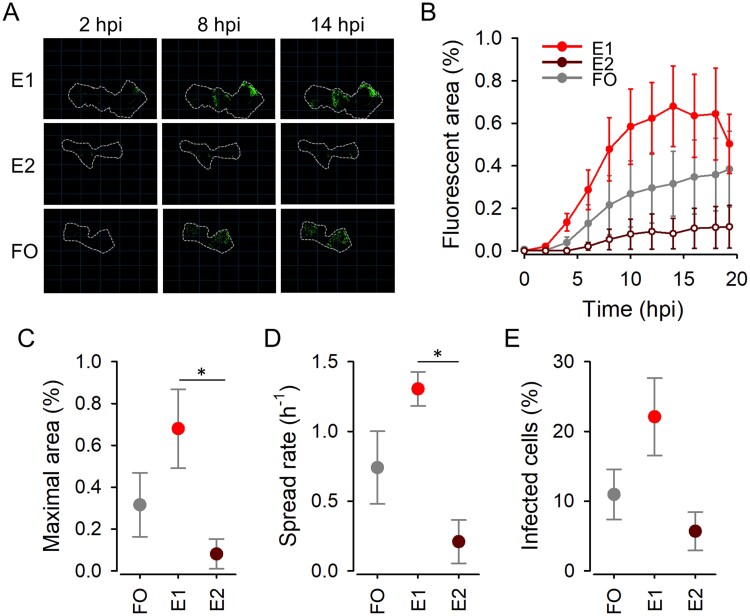
Viral fitness assays in explants. (A) Representative images of explants infected with each virus. Dashed lines indicate the contour of each explant. (B) Growth curves obtained by automated real-time fluorescence microscopy for E1–E2 and founder viruses. Lines are depicted to connect different time points only (no model fit). Error bars correspond to the SEM (*n* = 3 replicates). (C) Maximal observed infected area is plotted for each virus. (D) Viral spread rate, calculated as the log ratio between the fluorescent areas observed at 8 and 2 hpi. All explants were inoculated with 10^4^ PFU. Asterisks indicate significant differences between E1 and E2 (*t*-test: *P *<* *0.05). (E) Percent infected cells obtained by flow cytometry.

We also compared the two lines evolved in tumor explants (E1, E2) and the founder virus using real-time automated fluorescence microscopy. Although growth curves in explants showed high variability, as expected from *in vivo* samples, the E1 line appeared to spread faster and reach a higher fraction of cells than the founder virus based on direct imaging of the explants, whereas the E2 line did not seem to gain fitness (Fig. 4A–D). To better test this, we determined the percentage of infected cells at endpoint (22 hpi) after disaggregating the explants. Flow cytometry counts indicated that explants infected with E1 viruses showed a higher percentage of GFP-positive cells (22.1 ± 5.6 per cent) than E2 viruses (5.7 ± 2.4 per cent) or the founder (11.0 ± 3.8 per cent), although these differences did not reach statistical significance (*n* = 3; *t*-tests: *P* > 0.05; Fig. 4E). Therefore, evolution in explants yielded divergent results for the two lines and high variability in fitness assays.

### 3.3 Comparison of all evolved lines in monolayers

To investigate the specificity of adaptation to each of the culture systems, we compared the growth curves of the founder, M1–M4, S1–S4, and E1–E2 viruses in 4T1 monolayers in a single experimental block, using real-time automated fluorescence microscopy. As shown above, the spread rate of the M1–M4 viruses (ranging from *r* = 0.842 ± 0.090 to *r* = 1.117 ± 0.087) was approximately twice as that of the founder (*r* = 0.492 ± 0.010; *t*-tests: *P* < 0.019; Fig. 2D), and the maximal infected area was also significantly elevated in M1–M4 viruses (*t*-tests: *P* ≤ 0.020; Fig. 2D). The viruses evolved in spheroids (S1–S4) also tended to show higher *A*_max_ and *r* values than the founder, but lower than M1–M4 (nested ANOVA: *P* = 0.010 and *P* = 0.008 for *A*_max_ and *r*, respectively). For S1–S4 viruses, the change in fitness relative to the founder was similar when assayed in monolayers and spheroids, since both *A*_max_ and *r* were highest for S2 and S3, intermediate for S1 and S4, and lowest for the founder regardless of whether fitness was assayed in monolayers or spheroids (Fig. 2B and D). Concerning explant-evolved viruses, the spread rate of the E1 virus in monolayers was within the range of M1–M4 values and, hence, clearly higher than for the founder (*r* = 0.868 ± 0.020; *t*-test: *P* < 0.001; Fig. 2C and D). E1 also showed an *A*_max_ value similar to M1–M4 and higher than the founder virus (*t*-test: *P* = 0.006). In contrast, the fitness of the E2 virus was similar to that of the founder both in terms of *A*_max_ and *r*. Hence, the relative fitness values of E1 and E2 in monolayers mirrored the values obtained in explants.

### 3.4 Fitness assays in MEFs

**Figure 5.**
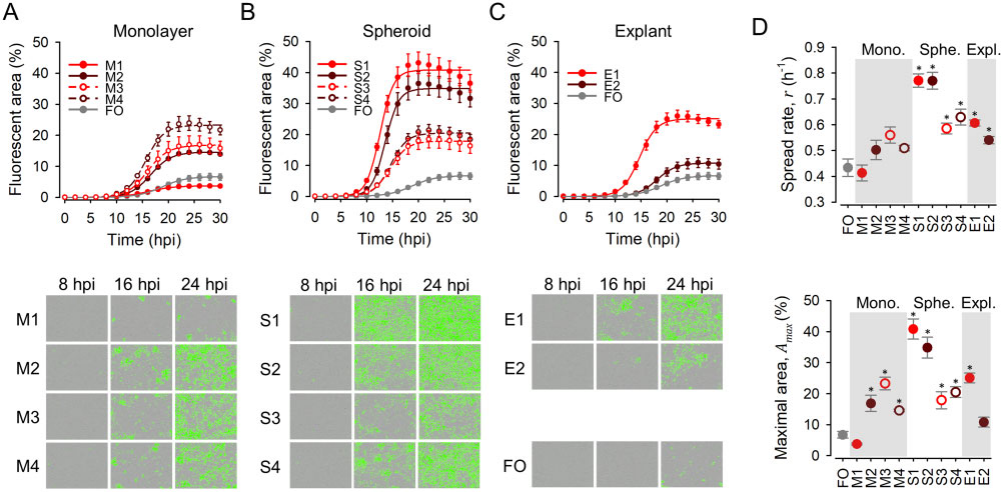
Viral fitness assays in MEFs. (A–C) Top: Growth curves obtained by automated real-time fluorescence microscopy for M1–M4 monolayer-evolved viruses, S1–S4 spheroid-evolved viruses, E1–E2 explant-evolved viruses, and the founder virus (FO), respectively. Cells were seeded in six-well plates at a density of 10^5^ per well and, after 24 h, cells were inoculated with approximately 10^4^ PFU. Lines indicate the predicted values obtained from a logistic growth model. Error bars correspond to the SEM (*n* = 3 replicates). Bottom: representative images obtained at different time points. (D) Spread rate (*r*) and maximal infected area (*A*_max_) obtained by fitting the logistic growth model to the data. Asterisks indicate values significantly different from that of the founder virus (*t*-test: *P *<* *0.05).

We then set out to test whether adaptation was cell type-specific. For this, we obtained growth curves for M1–M4, S1–S4, E1–E2, and the founder virus in monolayers of MEFs, a non-tumoral cell line. In the context of oncolytic viruses, this could help elucidate whether directed evolution in a tumor cell line (4T1) could concomitantly increase the ability of a virus to infect normal cells. We found that all lines evolved in 4T1 cells showed faster spread in MEFs than the founder virus, with the exception of M1 (Fig. 5 and Supplementary Fig. S3). Surprisingly, though, the best-adapted viruses in MEFs were those evolved in 4T1 spheroids, both in terms of *A*_max_ and *r*, particularly S1 and S2 (Tukey’s *post hoc* test: S1–S2 conformed a homogeneous group with significantly higher *A*_max_ and *r* than all other lines, *P* < 0.05). This contrasts with the growth assays performed in 4T1 monolayers, in which M1–M4 lines were superior to S1–S4 (Fig. 2). Therefore, viral adaptation to 4T1 cultures with different spatial structures influenced the ability to infect MEFs. Finally, the explant-evolved lines E1 and E2 showed similar fitness relative to the founder in both 4T1 and MEFs.

### 3.5 Evolution of IFN suppression

**Figure 6.**
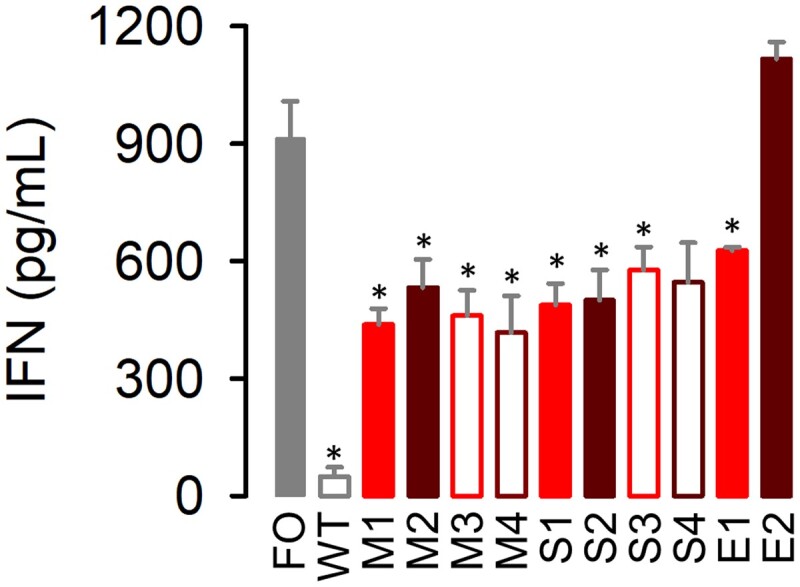
Interferon levels. IFN-β induced by the founder virus, the evolved lines, and a control WT virus in 4T1 monolayers. Error bars indicate the SEM (*n* = 3). Cells were inoculated with 3 PFU/cell and supernatants were collected at 16 hpi for IFN-β quantitation. Asterisks indicate values significantly different from that of the founder virus (*t*-test: *P *<* *0.05). The calibration curve is shown in Supplementary Fig. S4.

The 4T1 cell line is capable of eliciting an innate immune response against VSV (Andreu-Moreno and Sanjuán 2018). This should be an important factor limiting the fitness of the founder VSV-Δ51, since this mutant fails to block host gene expression and, hence, stimulates IFN-mediated antiviral responses (Stojdl et al. 2000, 2003). To test whether IFN suppression capacity was modified during the experimental evolution process, we measured IFN-β production by ELISA in 4T1 monolayers infected with the founder virus, M1–M4, S1–S4, E1–E2 or a virus that does not carry the Δ51 mutation (WT). We found that cells infected with all evolved lines except E2 showed significantly lower IFN-β production than those infected with the founder VSV-Δ51 virus, although IFN-β suppression was not complete, and less efficient than for the WT virus (*t*-tests: *P* < 0.013; Fig. 6 and Supplementary Fig. S4). Hence, the effects of the Δ51 mutation were partially compensated in the evolved lines. The inability of the E2 line to suppress IFN-β secretion could explain why this virus showed lower fitness than E1 (and similar to that of the founder) in 4T1 monolayers and explants, as well as in MEF monolayers.

To further test the importance of IFN suppression in these evolution experiments, we performed growth curves in mouse Neuro2a cells, which exhibit no ability to mount an antiviral innate immune response (Andreu-Moreno and Sanjuán 2018). Since this offered a more permissive environment, all tested viruses showed higher *A*_max_ and *r* values than in 4T1 or MEFs (Supplementary Fig. S5). Despite some statistically significant differences (M4, S2, E1, E2), the evolved lines showed small changes in *A*_max_ compared to the founder (≤5 per cent change, versus up 54 per cent in 4T1 cells and up to fivefold in MEFs). Changes in *r* values were larger, but still less pronounced than in 4T1 or MEFs. Antiviral innate immunity should have a stronger effect on the maximal infected area than on initial viral spread rate, since the IFN-mediated response is deployed after viral spread starts (Domingo-Calap et al. 2019). Hence, these results suggest that IFN suppression was an important selective factor driving the evolution of VSV-Δ51 in 4T1 cells, but not the sole factor.

### 3.6 Deep sequencing of the evolved lines

**Figure 7.**
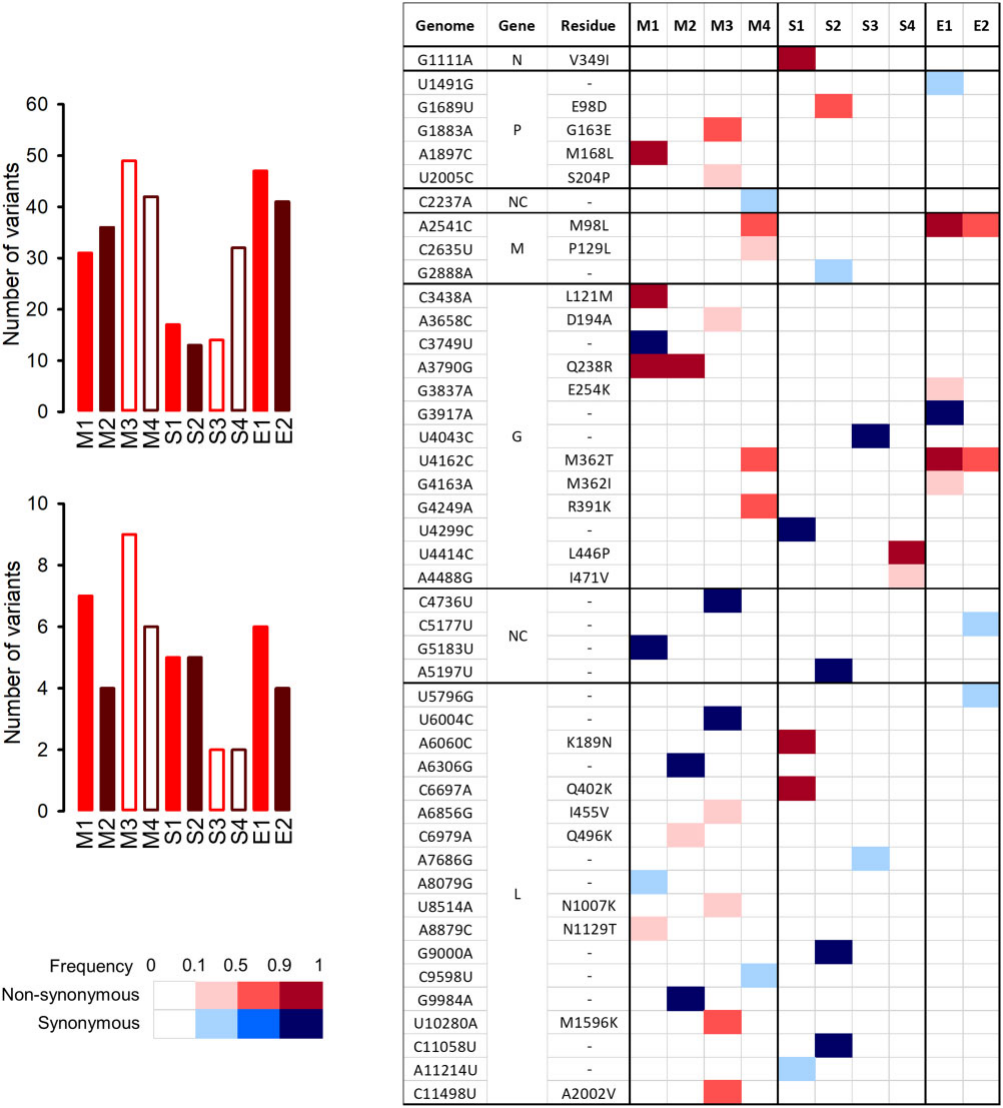
Sequence variants appeared in the evolved lines. Left: counts of sequence variants found at >1 per cent (top) or >10 per cent (bottom) population frequency in the evolved lines and not present in the founder. Right: Mapping of genetic variants found at >10 per cent frequency. The color legend indicates variant frequency and whether each mutation was synonymous (blue) or non-synonymous (red). Of these, G1111A, A3790G (in both M1 and M2 lines), U4043C, U4299C, U4414C, G5183U, A5197U, A6060C, C6697A, G9000A, G9984A, and C11058U reached frequencies >99 per cent.

We extracted RNA and performed Illumina sequencing to identify all sequence polymorphisms that reached a population frequency of 1 per cent or more in the evolved lines (Supplementary Table S1). The Δ51 deletion was preserved in all cases. Lines evolved in monolayers showed twice as many sequence variants (39.5 ± 3.9) as lines evolved in spheroids (19.0 ± 4.4; *t*-test: *P* = 0.013), whereas the two explant lines also showed relatively high numbers of variants (41 and 47; Fig. 7). Overall, only 15.5 per cent of these variants reached a population frequency of 10 per cent or more, and hence most probably did not produce detectable fitness changes at the population level. Above the 10 per cent frequency cutoff, the M1–M4 lines were still more diverse than S1–S4, although the lower counts reduced statistical power (6.5 ± 1.0 and 3.5 ± 0.9 variants, respectively; *t*-test: *P* = 0.069). Despite the fact that lines within each culture system evolved similarly (except E1 and E2) in terms of population fitness in monolayers, there were relatively few parallel evolution events at the sequence level (Fig. 7). The M98L amino acid replacement in the M protein was found in M4, E1, and E2 lines at >50 per cent population frequency, and the M362T replacement in the G protein was found in these same lines, suggesting some epistatic interaction between these two mutations. The other parallel evolution event was replacement Q238R in the G protein, which reached >90 per cent frequency in M1 and M2 lines. Spheroids showed no parallel substitutions.

## 4. Discussion

### 4.1 Similarities between viruses evolved in monolayers, spheroids, and explants

The data revealed some shared evolutionary patterns among culture systems. All lines except E2 showed evidence of adaptation to their respective environments, and the fitness changes experienced by lines evolved in 4T1 spheroids or explants were qualitatively reproduced when fitness was assayed in 4T1 monolayers. A shared selective pressure in the three culture systems was the antiviral innate immune response mounted by 4T1 cells, a factor that severely impairs viral spread, particularly during late infection. The observation that the founder and evolved viruses showed smaller fitness differences in Neuro2a cells supports the importance of IFN suppression as a factor driving viral evolution in the three 4T1 culture systems. The ability of the VSV-Δ51 virus to block this response improved in all lines except E2, potentially explaining why all evolved viruses (except E2) showed improved fitness compared to the founder virus. However, the IFN blockade showed by all evolved lines was always less efficient than that of WT VSV, indicating that the effect of the Δ51 was not fully compensated. We have previously shown that, albeit strongly beneficial for viral fitness, innate immunity evasion may not always be favored by natural selection. If variants capable of blocking IFN secretion are mixed with variants that do not block IFN secretion, the later exert a dominant negative fitness effect by stimulating innate immune responses in a paracrine manner (Garijo et al. 2016; Domingo-Calap et al. 2019; Segredo-Otero and Sanjuán 2020). This, together with the fact that genetic reversion of a three-base deletion such as Δ51 is unlikely, could explain why IFN suppression capacity improved only partially.

### 4.2 Differences between lines evolved in monolayers, spheroids, and explants

Evolutionary outcomes exhibited some differences depending on the culture system. First, lines evolved in spheroids were genetically less diverse than those evolved in monolayers, probably because infection was less efficient, reducing effective population sizes and, hence, the production of new genetic variants. Second, lines evolved in 4T1 spheroids tended to show higher fitness in MEFs that those evolved in 4T1 monolayers. MEFs are a less permissive cell line than 4T1. Specifically, VSV-Δ51 typically reaches titers on the order of 10^6^–10^7^ PFU/ml in MEF monolayers (Domingo-Calap et al. 2019), compared to 10^9^ PFU/ml in 4T1 monolayers. Although both MEFs and 4T1 mount an innate immune response against VSV-Δ51, MEFs are non-tumoral, metabolically less active, and more slowly proliferating cells. Limited access to nutrients or oxygen in spheroids compared to monolayers might reduce the metabolic activity of 4T1 cells. As a result, the intracellular environments of 4T1 spheroids and MEFs might share some similarities, potentially explaining the observed viral fitness correlations. Concerning explants, the E1 line was similar to M1–M4 lines in terms of adaptation, fitness in 4T1 monolayers, fitness in MEFs, and genetic diversity. In contrast, the E2 line showed poor performance in all assays, despite the fact that it harbored similar genetic diversity as E1 and M1–M4 lines, and shared two parallel mutations with M4 and E1.

### 4.3 Evolutionary repeatability

Within each culture system, replicate lines often produced different outcomes, revealing evolutionary stochasticity. First, M1 showed no fitness gain when assayed in MEFs, as opposed to M2–M4. Second, S1 and S2 showed higher fitness in MEFs than S3 and S4. Third, E1 and E2 followed markedly different evolutionary fitness trajectories in all assays performed, despite the fact that they both acquired mutations M98L and M362T. High among-line variability was also evident at the sequence level. Only three parallel mutations were found, and none appeared in more than three lines. This makes it difficult to identify adaptive mutations by looking at parallel evolution events. Previous work with VSV has revealed greater levels of parallel sequence evolution than those observed here in some cases, but not in others (Cuevas, Elena, and Moya 2002; Novella et al. 2004; Remold, Rambaut, and Turner 2008; Cuevas, Moya, and Sanjuán 2009; Garijo et al. 2014, 2016; Hernández-Alonso et al. 2015). The reasons for these differences might be attributed to effective population sizes (determined mainly by inoculum sizes), evolutionary time (number of transfers), and the strength of the selective pressures applied, among other possible factors (Sanjuán and Grdzelishvili 2015). Although evolutionary repeatability was relatively low, some interesting parallel evolution events can be identified by comparing across studies. First, the E254K substitution in gene G, which appeared in line E1, was previously reported in 4/5 WT lines evolved in MEFs (Hernández-Alonso et al. 2015). Interestingly, this mutation was shown to be deleterious when assayed individually by site-directed mutagenesis, suggesting epistasis with other mutations. A more recent study also reported the E254K substitution in a human pancreatic ductal adenocarcinoma cell line, where it improved VSV cell surface attachment (Seegers et al. 2020). Other changes at this residue, such as E254Q and E254G, have also been reported (Janelle et al. 2011). Second, previous work showed that certain changes outside the M protein could compensate for the Δ51 defect. Specifically, substitutions at residue 168 of the P protein were shown to restore IFN suppression capacity in VSV-Δ51 experimentally evolved in MRC-5 human cells (Garijo et al. 2016). This same residue was mutated in our M1 line. Substitution P129L in the M protein, which was found in the M4 line, was also reported for VSV-Δ51 evolved in MRC-5 cells (Garijo et al. 2016). However, it is noteworthy that, despite 9/10 lines improved IFN suppression capacity, we found no shared genetic basis for this phenotypic change.

### 4.4 Implications for oncolytic virus evolutionary optimization

Differences in selective pressures *in vivo* and in standard cell cultures could result from cellular metabolic activity levels, the presence of a spatial structure limiting viral particle diffusion, the extracellular matrix, cancer-associated cell types, and immune responses, among other factors. For instance, since tumors contain mixes of different cell types, including fibroblasts, viral fitness, and oncolytic efficacy *in vivo* might depend on the ability of the virus to also infect these cells, which might otherwise act as ‘firewalls’ blocking viral spread. However, explant-evolved lines did not show greater ability to infect MEFs than lines evolved in pure 4T1 cultures. In principle, directed evolution in standard cell cultures (monolayers) might fail to capture important selective pressures present *in vivo*. Hence, it might not be viewed a priori as the best approach for oncolytic virus optimization. One could expect that the most efficacious viruses in a given environment should be obtained by carrying out the evolution in this same environment. However, this should not necessarily be the case. Relevant selective pressures, such as for instance receptor-dependent cell entry or innate immunity evasion, should be present even in the simplest culture systems, making them relevant for optimizing a virus *in vivo*. Viruses evolved in simplified systems could even outperform those evolved in more complex and biologically relevant systems because the former tend to be more permissive for viral replication, allowing the virus to reach higher population sizes, to reduce generation times and, consequently, to adapt faster under the action of selection. *In vivo* testing would be needed to conclusively show which culture system (monolayers, spheroids, or explants) best optimizes oncolytic activity. However, based on our results, evolution in monolayers appeared to yield more efficient and reproducible adaptation and higher cell-type selectivity than evolution in spheroids or explants.

One could as well envisage optimizing oncolytic viruses directly by *in vivo* evolution. This has been rarely attempted, and in nearly all cases, very short episodes or viral replication in nude mice (which also represent a simplified system) were alternated with multiple rounds of replication in cell cultures or embryonated eggs (Gros et al. 2008; Beier, Hermiston, and Mumberg 2013). Interestingly, in some cases the evolved virus tended to be less fit in cell monolayers than the parental virus. Although the genetic basis of adaptation was not elucidated, the fact that evolved viruses formed large cell syncytia suggested that cell-to-cell spread was favored in tumors (Beier, Hermiston, and Mumberg 2013). In general, *in vivo* infections are expected to yield poorer replication than cell culture systems and, hence, slower adaptation, as well as higher variability among lines, in addition to technical and ethical issues. Due to these limitations, we did not undertake VSV-Δ51 optimization *in vivo* here.

## Supplementary Material

veab045_Supplementary_Data
