# Supplementary material for: Experimental virus evolution in cancer cell monolayers, spheroids, and tissue explants
Source: Virus Evol. 2021 May 6;7(1):veab045. doi: 10.1093/ve/veab045 (PMC8134955; doi:10.1093/ve/veab045)
Supplement: veab045_Supplementary_Data [file veab045_supplementary_data.zip › AlZaher_supplementary.docx]

**
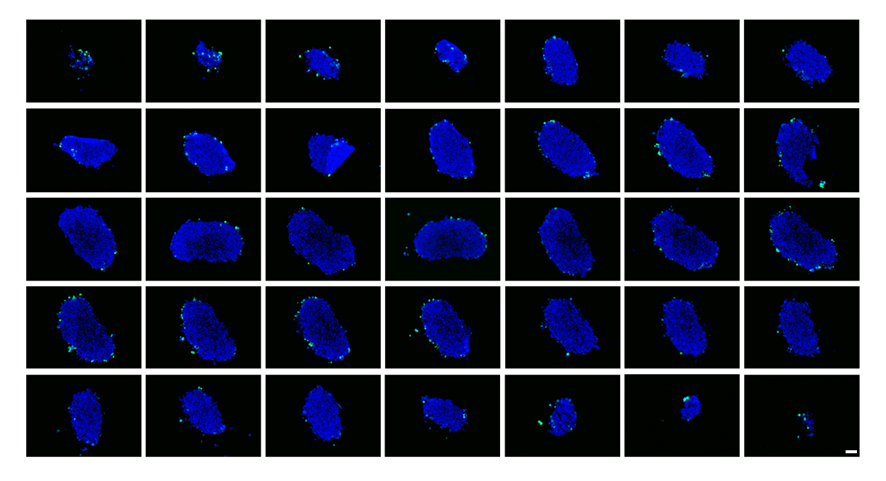
Supplementary material**

**Figure S1. Serial sections of a spheroid infected with VSV-Δ51-GFP.** Sections were prepared with a cryostat and stained with DAPI to show cell nuclei. The infection was essentially restricted to the spheroid surface. Bar: 75 µm.


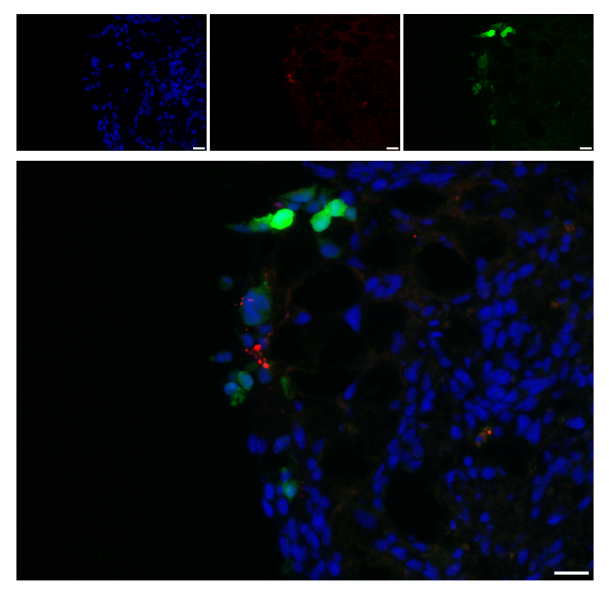


**Figure S2. Section of an explant infected with VSV-Δ51-GFP.** Top left: DAPI. Top center: Cytotox (a cell death marker). Top right: GFP. Bottom: merge. Bar: 25 μm.

**
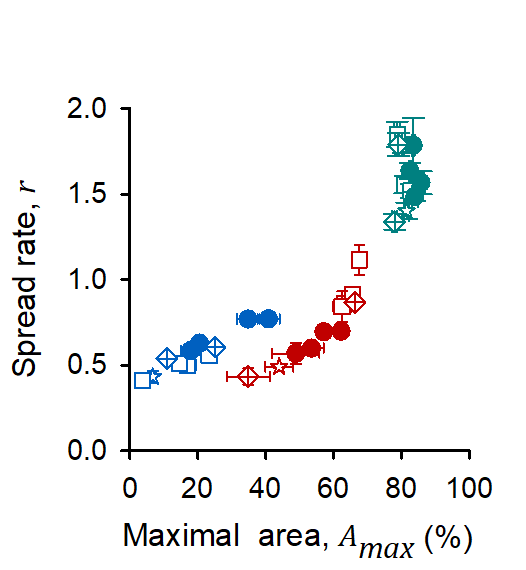
**

**Figure S3. Maximal infected area and spread rate inferred from the logistic model.** Parameters are as shown in **Fig. 2**, **Fig. 5**, and **Fig. S5.** Blue: MEFs. Red: 4T1. Green: Neuro2a. Stars: founder. Squares: M1-M4 lines. Filled circles: spheroids. Crossed diamonds: explants. Error bars represent the SEM (n = 3).


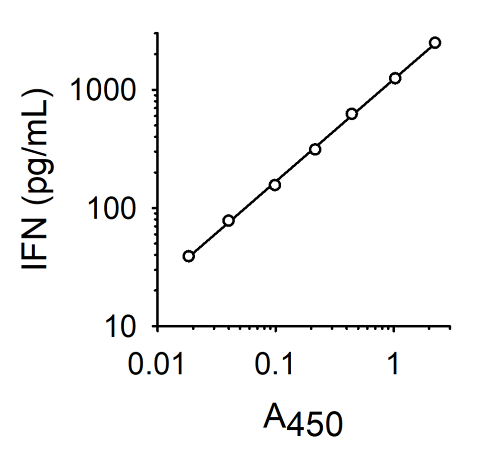


**Figure S4. Calibration curve for IFN-β quantitation by ELISA.** The log-scale least-squares regression is shown.


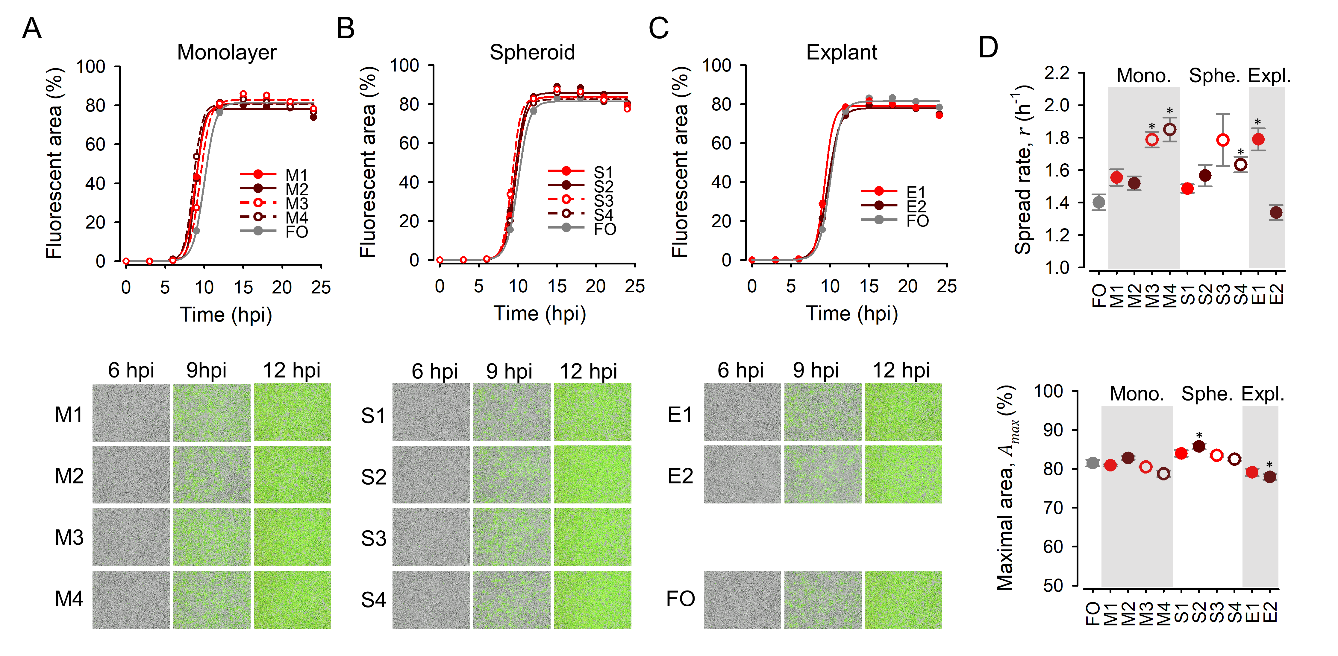


**Figure S5. Viral fitness assays in Neuro2a monolayers. A, B, C.** Top: Growth curves otained by automated real-time fluorescence microscopy for M1-M4 monolayer-evolved viruses, S1-S4 spheroid-evolved viruses, E1-E2 explant-evolved viruses, and the founder virus (FO), respectively. Cells were seeded in 6-well plates at a density of 10^5^ per well and, after 24 h, cells were inoculated with approximately 10^4^ PFU. Lines indicate the predicted values obtained from a logistic growth model. Error bars correspond to the SEM (n = 3 replicates). Bottom: representative images obtained at different time points. **D.** Spread rate (*r*) and maximal infected area (*A_max_*) obtained from the logistic growth model. Asterisks indicate values significantly different from that of the founder virus (t-test: *P* < 0.05).

**Table S1. Sequence variants found at >1% frequency in the founder and evolved populations by deep sequencing (Excel format).**
