# Supplementary figures and images for: Experimental virus evolution in cancer cell monolayers, spheroids, and tissue explants
Source: Virus Evol. 2021 May 6;7(1):veab045. doi: 10.1093/ve/veab045 (PMC8134955; doi:10.1093/ve/veab045)

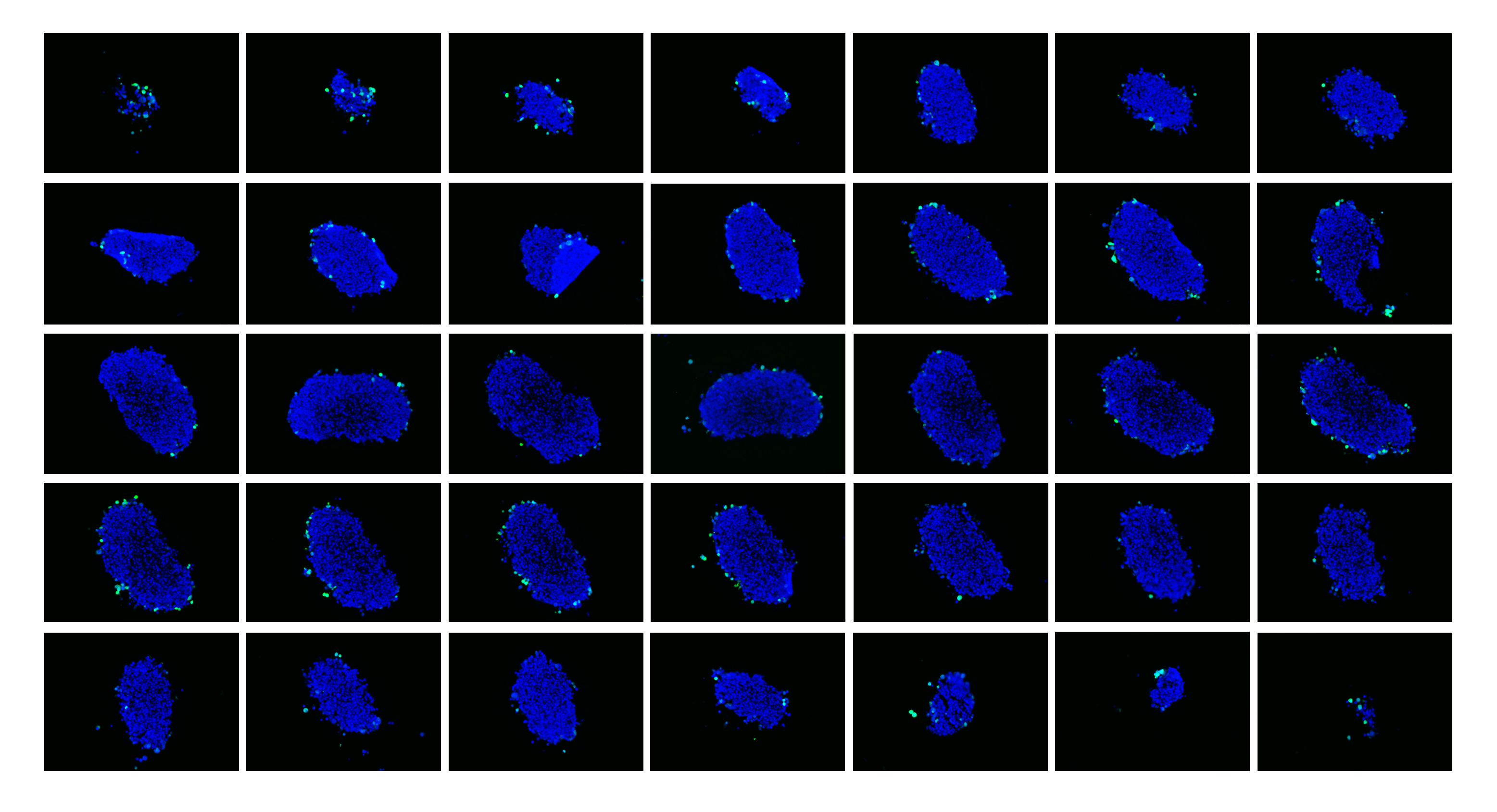

Supplement: veab045_Supplementary_Data [file veab045_supplementary_data.zip › FigS1.TIF]

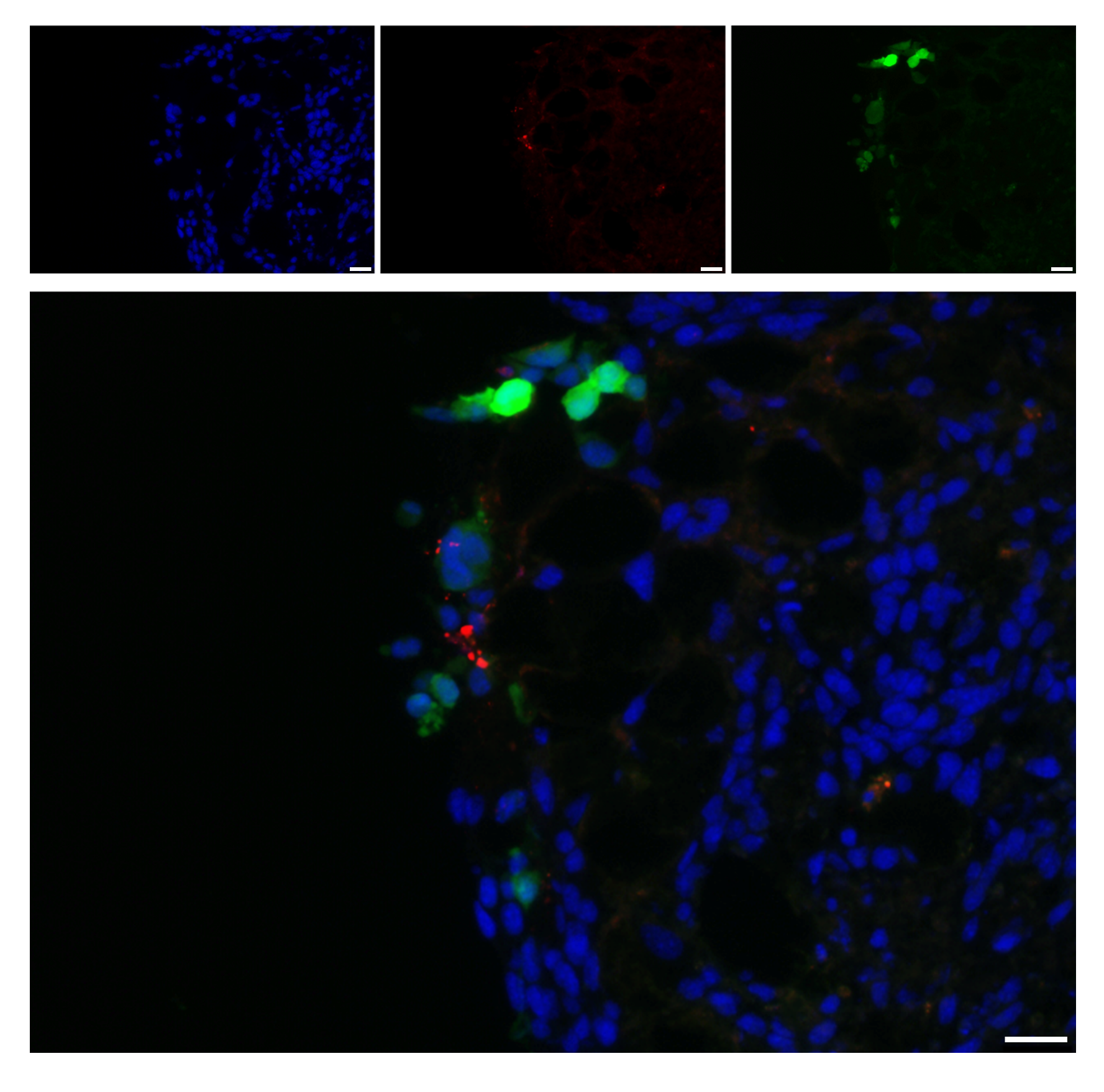

Supplement: veab045_Supplementary_Data [file veab045_supplementary_data.zip › FigS2.TIF]

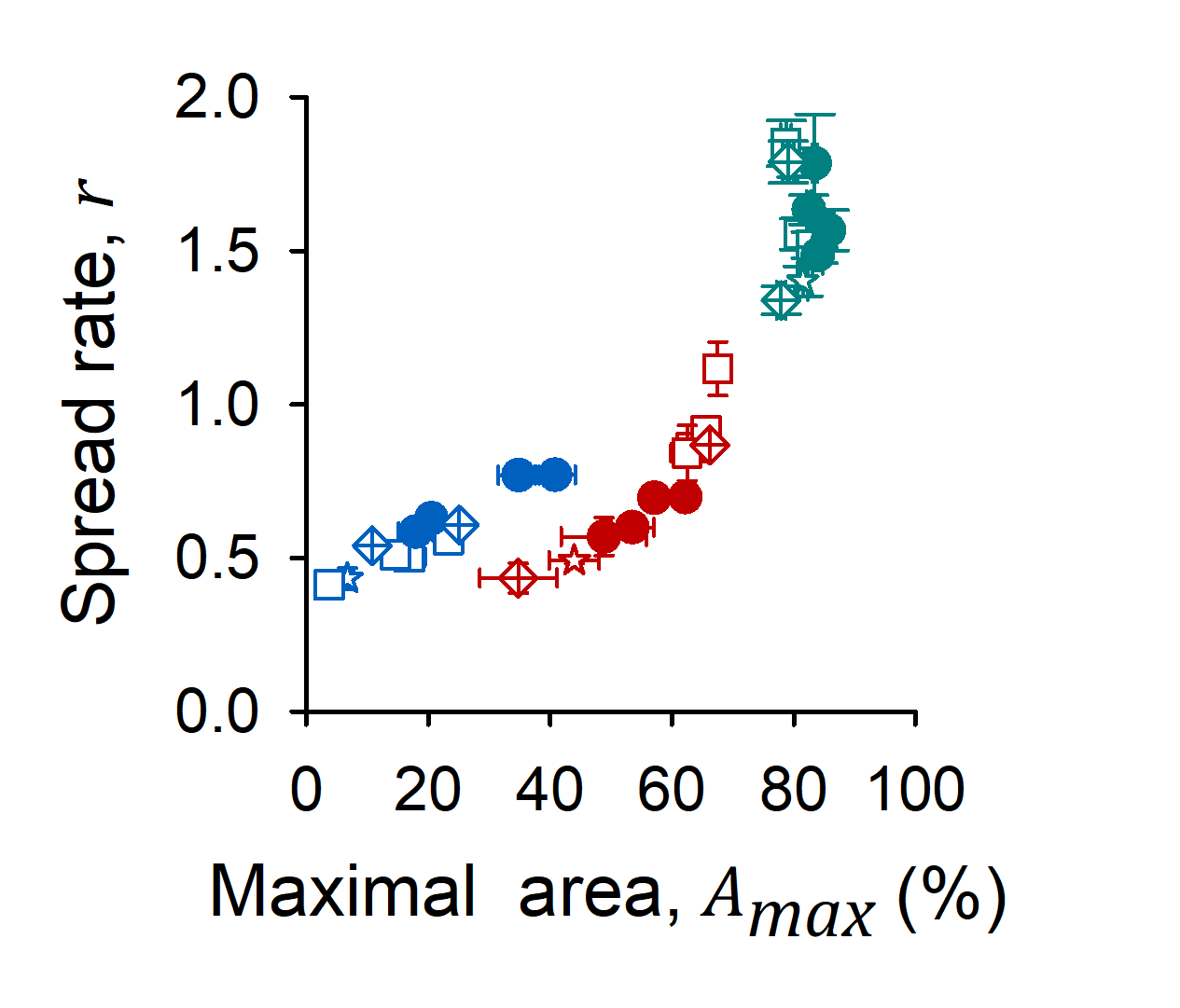

Supplement: veab045_Supplementary_Data [file veab045_supplementary_data.zip › FigS3_v2.tif]

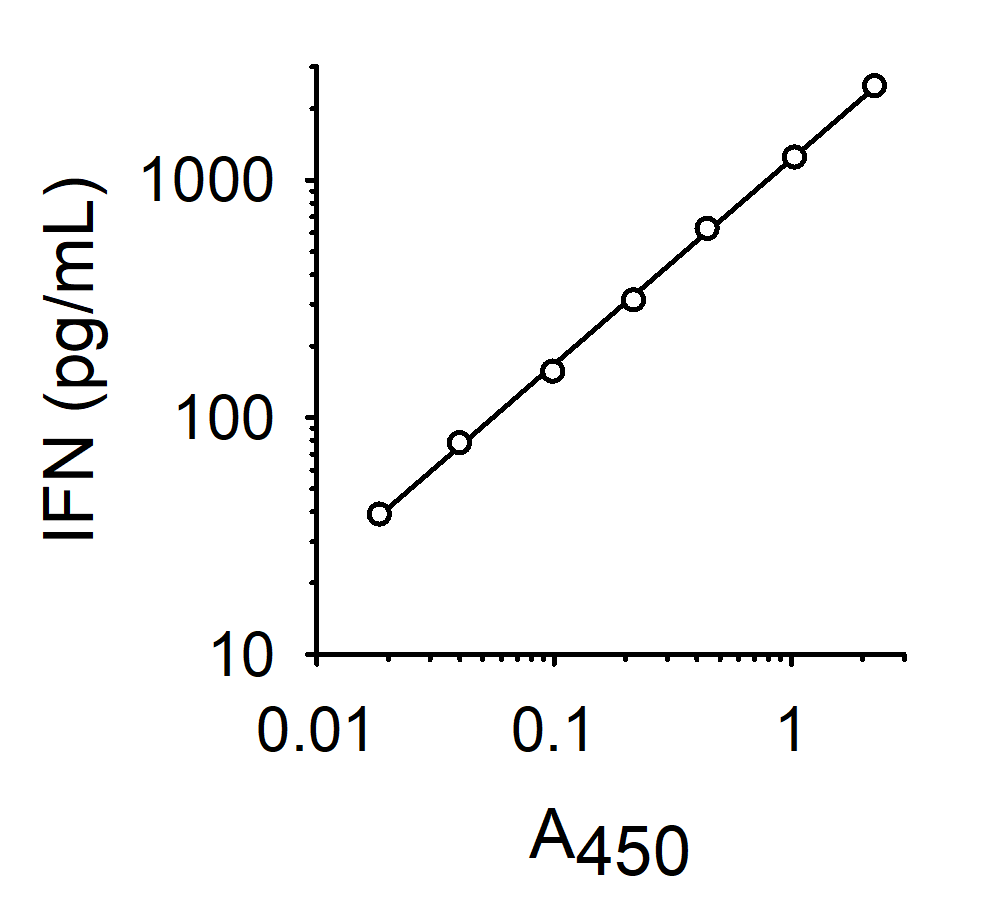

Supplement: veab045_Supplementary_Data [file veab045_supplementary_data.zip › FigS4.tif]

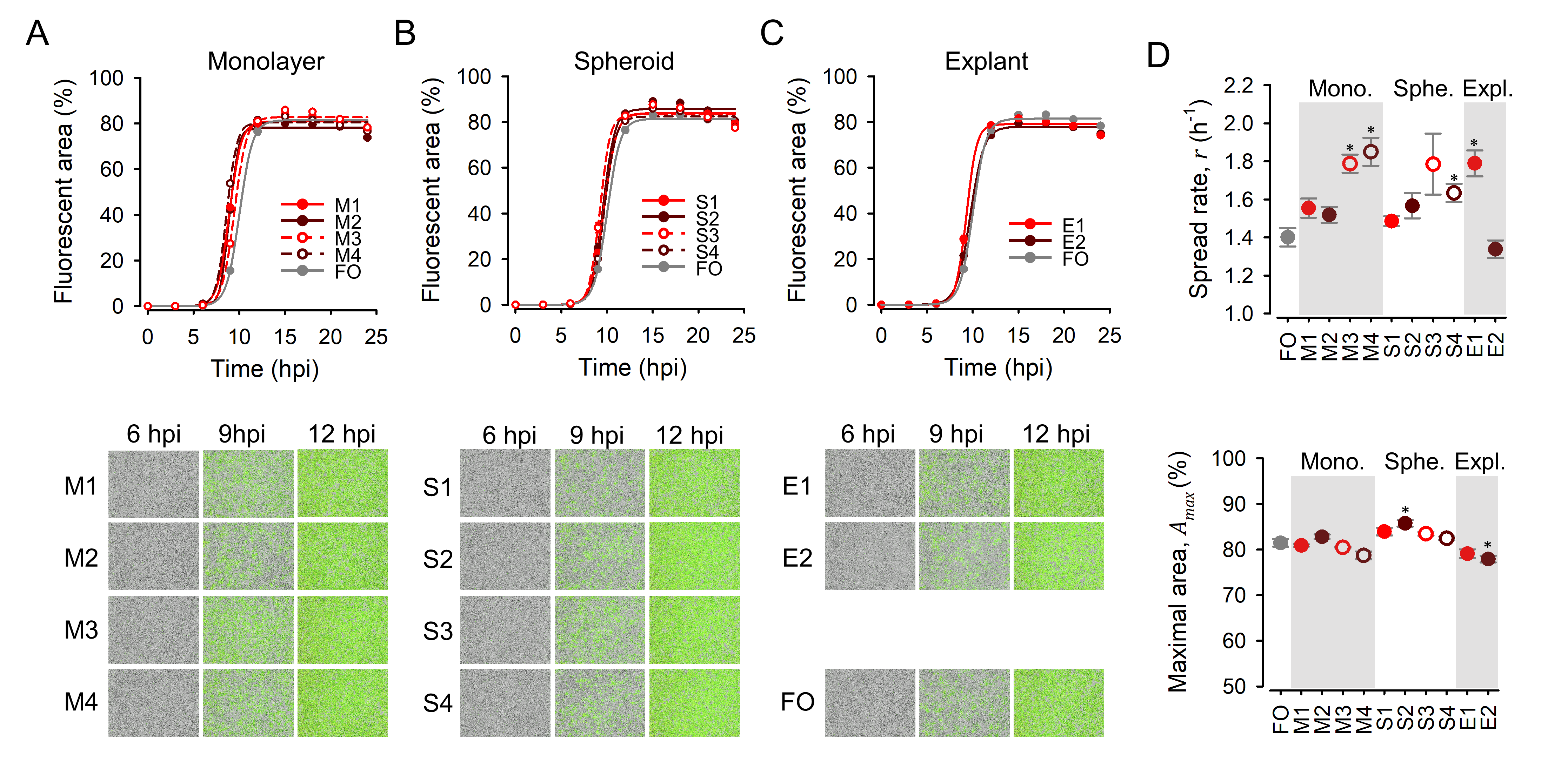

Supplement: veab045_Supplementary_Data [file veab045_supplementary_data.zip › FigS5_v2.tif]
